# Computational Assessment of Electrical and Thermal Effects of Epicardial Pulsed Field Ablation Adjacent to Stented Arteries

**DOI:** 10.3390/bioengineering13070825

**Published:** 2026-07-17

**Authors:** Francisco Estevez-Laborí, Maite Izquierdo, Ken Coffey, Barry O’Brien, Ana González-Suárez

**Affiliations:** 1 BioMIT, Department of Electronic Engineering, Universitat Politècnica de València, Building 7F, Camino de Vera, s/n, 46022 Valencia, Spain; festlab@doctor.upv.es; 2 Electrophysiology Unit, Cardiology Department, Hospital Universitario y Politécnico La Fe de Valencia, 46026 Valencia, Spain; maiteizqui@hotmail.com; 3 AtriAN Medical Ltd., Unit 204, University of Galway Business Innovation Centre, Upper Newcastle, H91 W60E Galway, Ireland; ken.coffey@energypulsed.com (K.C.); barry.obrien@atrianmedical.com (B.O.)

**Keywords:** computer modeling, coronary artery, metal stent, pulsed field ablation, thermal-side effects

## Abstract

Background: Current ablation strategies for the treatment of cardiac arrhythmias remain suboptimal. Treating cardiac arrhythmias using epicardial pulsed field ablation (PFA) selectively targets ganglionated plexi (GPs) within epicardial fat, offering a promising alternative to thermal ablation. Previous computational studies lacked physiological realism, excluding catheter geometry, fluid flow and post-PFA thermal latency. This study aimed to develop a realistic 3D epicardial PFA model integrating a clinical catheter, clinical PFA parameters and a sequentially coupled electro-thermal-fluid dynamics model, including thermal latency, to assess electrical and thermal collateral effects near stented coronary arteries. Methods: The model included epicardial fat, myocardium, blood, and the left circumflex artery containing a metallic stent positioned 0.25 mm beneath the catheter electrodes. Pulses of 1000, 2000, and 2500 V (60 pulses × 100 µs, 1 Hz) were simulated to analyze electric field distribution, PFA-induced lesion volume, temperature evolution, and Arrhenius-based thermal damage, including a 90 s post-pulse period to account for thermal latency. The PFA-threshold of 1000 V/cm was considered. Results: The artery reduced PFA-induced lesion size mainly by occupying fat tissue volume, while the stent shielded the lumen without altering fat lesion volume. The presence of a stent produced localized electric field enhancement at the arterial wall, with up to 3.83% of the arterial wall volume affected by PFA in the worst-case configuration. At clinical settings (1000 V), temperature remained below 40 °C and no collateral damage occurred. Voltages > 2000 V increased arterial wall heating, with thermal damage expanding up to five-fold during latency in the epicardial fat. Myocardium remained unaffected in all cases. Conclusions: The computational model developed in this study indicates that clinically relevant PFA parameters (1000 V) produce localized electric field enhancement at the stent–artery interface, resulting in limited collateral electrical effects in the arterial wall, while avoiding collateral thermal effects and preserving the myocardium. However, the use of higher pulse voltages can lead to delayed thermal damage within the epicardial fat.

## 1. Introduction

**Figure 1:**
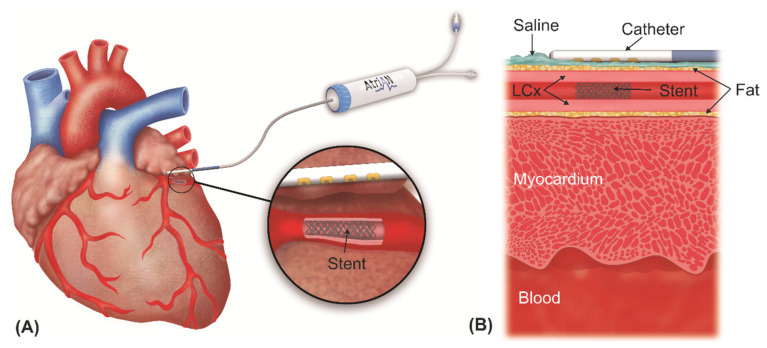
Physical setup during epicardial pulsed field ablation (PFA). (**A**) The Finger device (AtriAN Medical, Galway, Ireland) is placed on the left atrium epicardial surface with the left circumflex coronary artery (LCx) situated just below the ablation point. (**B**) The simulated worst case corresponded with the one in which a metal stent was placed inside the LCx just beneath the electrodes.

Pulsed field ablation (PFA) is an emerging treatment for cardiac arrhythmia. Despite its growing application, current treatment strategies remain suboptimal. Traditionally, catheter ablation techniques have focused on pulmonary vein isolation by creating circumferential lesions, using either thermal energy sources such as radiofrequency (RF) [1,2,3] or non-thermal energies such as PFA [4,5]. Alternatively, epicardial PFA can selectively ablate the ganglionated plexi (GPs), an extensive neuronal network with groups of ganglia interconnected by epicardial axons and typically embedded in epicardial fat [6]. This tissue selectivity allows PFA to spare other tissues, such as healthy myocardium. The treatment approach for epicardial PFA involves positioning a catheter over the epicardial surface to apply high-voltage pulses to target the epicardial fat where the GPs are embedded, as illustrated in Figure 1.

Substantial preclinical evidence and initial clinical results have suggested that epicardial PFA is a safe and effective treatment for cardiac arrhythmias [7,8]. However, recent preclinical and clinical findings have revealed that pulmonary vein isolation using PFA, although fundamentally non-thermal and tissue-selective, can induce transient coronary artery vasospasm when applied near epicardial vessels [9,10]. Acute, reversible coronary constriction has been consistently documented in both animal and human studies, typically resolving within minutes following the administration of nitroglycerin [11]. While these vasospastic events rarely result in lasting arterial injury or ischemia, histological analyses have identified focal medial fibrosis and mild intimal hyperplasia in some cases [12,13]. These findings underscore that, despite the inherent selectivity of PFA, electric fields interacting with vascular smooth muscle cells may transiently alter vascular tone through calcium-dependent mechanisms [12].

The ideal treatment approach would achieve selective ablation of GPs without damaging the myocardium or causing secondary side effects that could complicate treatment and post-procedural recovery. Achieving this selectivity is essential to properly assess the anti-arrhythmic potential of epicardial PFA. In this context, computational modeling has previously been used to predict electric field distribution in the various tissues involved (fat, myocardium, and blood), while coupled electro-thermal simulations have enabled quantification of thermal side effects associated with high-voltage pulses. Additionally, the potential disruptive electrical and thermal effects of nearby stented arteries have also been explored through simulation studies [14,15,16].

However, these previous models overlooked several critical aspects that significantly limit the clinical applicability of their findings. First, they did not model the prototype catheters (Finger and Glove) developed by AtriAN Medical Ltd. (Galway, Ireland) for epicardial PFA [17], nor did they consider the PFA clinical treatment parameters in terms of pulse duration and separation between pulses: a train of 10 pulses, each 100 µs in duration with 100 µs inter-pulse intervals, was used instead of clinical trial settings of a 60-pulse train with identical pulse duration but 1 s inter-pulse intervals. Second, they omitted the fluid dynamics in the heat exchange equations, which is crucial to realistically modeling the thermal effects of blood flow in the cardiac chamber and within the coronary artery (when present). Third, thermal latency, the continued heat buildup and potential tissue damage occurring after the PFA protocol has finished, was not considered. Finally, to date, the potential collateral effects of PFA on the arterial wall have not been systematically investigated. These limitations reduce the clinical relevance of earlier results and underscore the need for a more comprehensive and physiologically accurate computational model.

Given the concerns associated with PFA in proximity to a coronary artery and the limitations of previous models, we considered the most critical scenario, in which an epicardial PFA model was developed with the catheter placed on the epicardial surface very close to a coronary artery, including an explicitly modeled coronary stent. This modeling approach incorporates the following features: (1) integrating the clinical trial catheter design (Finger device, AtriAN Medical, Galway, Ireland) and the clinical treatment parameters, (2) including a sequentially coupled multiphysics formulation in which the steady-state blood and saline flow fields were first computed and subsequently incorporated into the transient electro-thermal analysis, and (3) taking the accumulated thermal damage due to thermal latency into account. The computer simulations were planned to explore the effects of energy dosing and to estimate the efficacy and possible electrical- and thermal-side effects of epicardial PFA in the presence of a stented artery, a scenario practically impossible to evaluate with precision in preclinical and clinical settings.

To the best of our knowledge, this is the first computational study to simultaneously integrate all these critical factors. The insights gained from this work may support broader clinical adoption of epicardial PFA for the treatment of cardiac arrhythmias and contribute to enhancing the safety and effectiveness of this therapeutic approach.

## 2. Materials and Methods

### 2.1. Physical Situation and Model Geometry

Figure 1A shows the physical setup during epicardial PFA, in which the Finger device (AtriAN Medical, Galway, Ireland) is placed on the left atrium epicardial surface with the left circumflex coronary artery (LCx) situated just below the ablation point. We considered a situation in which a metal stent was placed inside the LCx just beneath the electrodes, representing the worst-case scenario possible in terms of electrical and thermal interaction (see Figure 1B).

**Figure 2:**
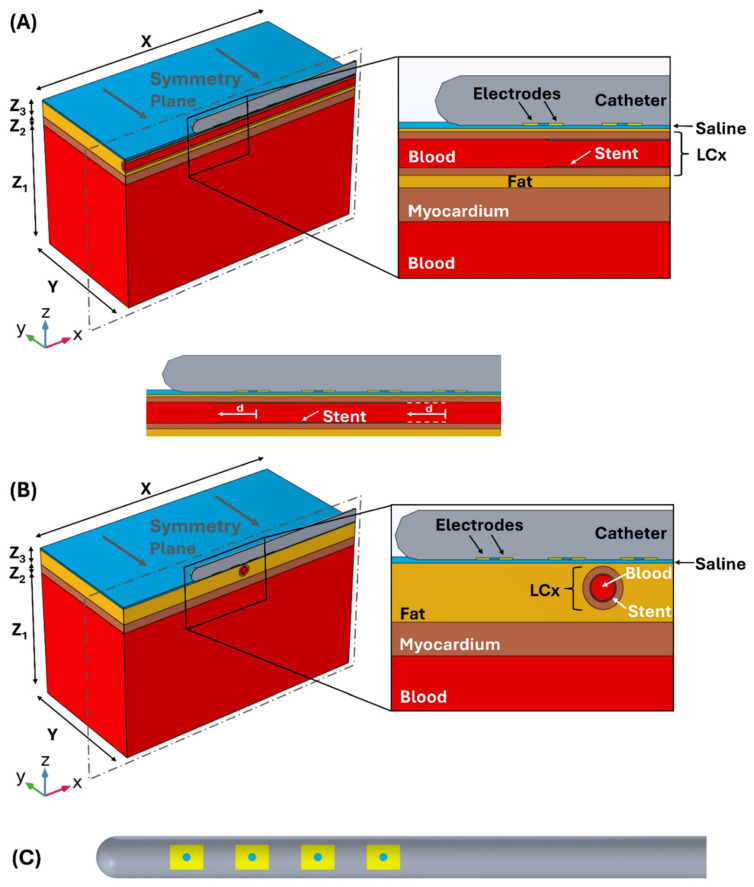
Geometry of the computational models with the LCx in a parallel (**A**) and perpendicular (**B**) orientation with respect to the catheter (X = 80 mm, Y = 40 mm, Z_1_ = 40 mm, Z_2_ = 2.7 mm, Z_3_ = 4.75 mm). The stent was displaced with an offset distance (d) of 3 mm at each step from its initial position directly beneath the electrodes to the left side of the LCx. (**C**) Detail of the epicardial PFA catheter: the four yellow rectangles represent the metal electrodes, each featuring central lumen (shown in blue) through which saline is infused.

Figure 2 shows the 3D limited-domain computational models used in this study. An XZ symmetry plane was applied through the center of the catheter and across the different tissue layers to reduce computational cost. The feasibility of using a limited-domain model has been previously validated through comparison with a full torso model [18]. The geometry included both the PFA catheter and a fragment of the relevant tissues: epicardial fat, myocardium and blood. Since the catheter comprised irrigated electrodes, a thin layer of saline (0.5 mm thick) was assumed to cover the epicardium surface around the catheter. This layer acts as a ‘virtual electrode’ to facilitate the transmission of electric energy into the target tissue [19]. A fragment of the coronary artery and a metal stent were also considered in some simulations.

Although the targets of epicardial PFA are indeed the GPs embedded in the epicardial fat, their size is extremely small compared to the other tissues (mean area in humans of 0.07 ± 0.02 mm^2^) [20], and for this reason they were not considered as subdomains in the model. This simplification does not affect the overall results in terms of boundary of tissue affected by electric field, as field distortion is exclusively limited to the surroundings of the GPs [20,21]. This exclusion also allows us to assume that its impact on the global temperature distribution will be minimal.

The Finger catheter (AtriAN Medical Ltd., Galway, Ireland) was modeled with all the realistic details: 3.98 mm in diameter and four metal electrodes of 3.18 mm in length separated by 3.05 mm with a central hole of 0.76 mm in diameter for saline infusion. The metal electrodes were assumed to be in direct contact with the epicardial surface and inserted 0.25 mm in the saline layer, which is equivalent to a low contact force [21]. Average anatomical dimensions for the tissue layers in the adult population were considered in the model: 4.75 mm for the epicardial fat layer and 2.7 mm for the myocardium [22,23]. The LCx diameter was 2.3 mm (which is within the reported anatomical range of 1.9–2.7 mm) [24] with a wall thickness of 0.6 mm (value reported in patients with atherosclerosis) [25] and at 0.25 mm beneath the catheter [14]. The stent was modeled as a hollow metal cylinder with a 100 µm wall and 20 mm length (which is in the range of 8–38 mm stent lengths for vessels ~2.25 mm) [26]. The stent was assumed to be initially positioned just beneath the two central metal electrodes (representing the worst-case scenario) and then horizontally displaced by an offset distance (d) of 3 mm at each step (Figure 2A). Both parallel and perpendicular orientations of the LCx relative to the catheter were considered, as shown in Figure 2A and Figure 2B, respectively.

### 2.2. Governing Equations

The 3D computational models solved a sequentially coupled electro-thermal-fluid dynamics problem. This was implemented numerically using the Finite Element Method (FEM) with COMSOL Multiphysics 6.2 (COMSOL Inc., Burlington, MA, USA). The simulations were conducted in two sequential steps.

**Figure 3:**
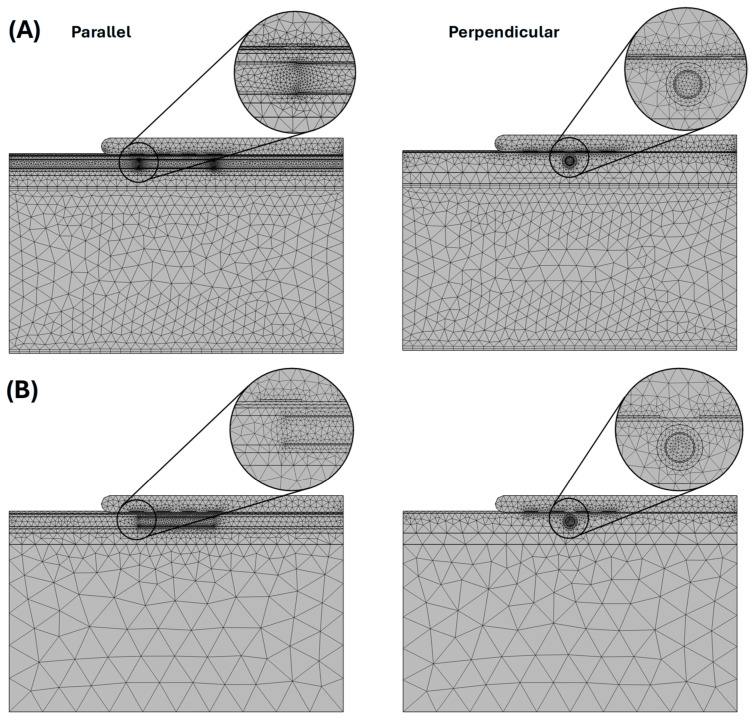
Physics-optimized meshes used to solve the fluid dynamics problem and the coupled electric–thermal problem. (**A**) Meshes employed for the fluid dynamics simulations for the LCx artery in parallel and perpendicular orientations. (**B**) Meshes used to solve the coupled electric–thermal problem for both LCx orientations. Due to the very thin stent wall, local mesh refinements were required in and around the stent region, particularly for the mesh used to solve the intraluminal flow dynamics. Detailed views of these refinements are shown in the magnified insects highlighted by the black circles.

The first step of the computational process aimed to solve the flow velocity ***u*** (m/s) for blood and saline under steady-state conditions, using a stationary solver. These velocity fields were then imported into the transient electro-thermal model, where the electrical and thermal equations were solved in a coupled manner while treating the flow field as fixed throughout the pulse sequence. A physics-controlled mesh was employed, consisting of 177,483 elements and 46,917 nodes for the parallel orientation, and 57,566 elements and 13,840 vertices for the perpendicular orientation (see Figure 3A).

This step utilized the incompressible Navier–Stokes equations to enforce conservation of momentum and mass:

(1) $$\rho \frac{\partial u}{\partial t}+\rho u\cdot \nabla u=-\nabla P+\mu \nabla^{2}u+F$$

(2) ∇·u=0 

where μ is the dynamic viscosity of blood (Pa·s), *P* is the pressure (Pa), and ***F*** the body forces (N/m^3^) which were neglected in our model.

The velocity field ***u*** obtained from the first step was used as an input parameter to the second phase of the simulation, enabling the solution of the coupled electrical-thermal problem using a time-dependent solver. Because the temperature rise during clinically relevant PFA is limited and does not significantly alter blood or saline flow, feedback from the thermal solution to the fluid dynamics was neglected. An optimized mesh of 68,508 elements and 13,821 vertices for the parallel orientation and 65,714 elements and 13,007 vertices for the perpendicular orientation (Figure 3B) was used to accurately resolve the fine structure of the stent and the surrounding interface regions. Due to the small thickness of the stent wall, local mesh refinement was required in this region.

Transient cellular responses, such as membrane charging, were assumed to be negligible and therefore were not included in the model. Consequently, a quasi-static approximation was used to compute the electric field distribution by solving Maxwell’s equations in its Laplacian form [27]:

(3) ∇⋅(σ∇ϕ)=0

(4) E=−∇ϕ

(5) J=σE

where σ is the electrical conductivity of the material, ϕ is the electrical voltage, E is the electric field vector, and J is the current density vector. The thermal problem was included in evaluating potential thermal side effects during PFA treatment. This was addressed by solving the Pennes’ Bioheat transfer equation [28]:

(6) $$\rho C_{p}\frac{\partial T}{\partial t}+\rho C_{p}u\nabla T=\nabla \cdot (k\nabla T)+Q+Q_{p}+Q_{met}$$

where ρ is density (kg/m^3^), $C_{p}$ is specific heat (J/kg·K), u is the flow velocity field (m/s) solved in the first computation step, *T* is temperature (°C), *t* is time (s), *k* is thermal conductivity (W/m·K), *Q* is the heat source caused by the electrical power associated with PFA (W/m^3^), *Q_p_* is the heat loss caused by blood perfusion (W/m^3^) and *Q_met_* is the metabolic heat generation (W/m^3^). Both *Q_met_* and *Q_p_* were ignored as these terms are negligible compared to the others [28], especially for short-duration heating [29].

### 2.3. Material Properties

The physical properties of the materials of the different elements of the model are shown in Table 1.

The electrical conductivity σ of the treated tissue changes dynamically in response to the local electric field, as cells become increasingly permeabilized due to PFA-induced pore formation. In addition, increasing tissue temperature further increases conductivity. Consequently, tissue electrical conductivity was modeled as a function of both the local electric field and temperature, following previously established approaches [34,35,36], as follows:

(7) $$\sigma (E,T)=\sigma_{0}\left[1+A\cdot flc2hs(E-E_{del},E_{range})+\alpha \;(T-T_{0})\right]$$

where $\sigma_{0}$ is the initial (pre-electroporation) conductivity; A is the conductivity increase factor after electroporation (i.e., $\sigma_{1}=\sigma_{0}\cdot \left(1+A\right)$), where $\sigma_{1}$ is the post-electroporation conductivity); flc2hs represents the smoothed Heaviside step function as implemented in COMSOL; $E_{del}$ is the midpoint of the transition zone; and $E_{range}$ defines the width of the transition zone. The parameter α (1/°C) is the temperature coefficient of conductivity, and $T_{0}$ is the initial tissue temperature (37 °C). A value of α=+2%/°C was used [37].

The pre- and post-electroporation conductivities ($\sigma_{0}$ and $\sigma_{1}$, respectively) were considered equivalent to the values measured at 10 Hz and 500 kHz, as justified in [35]. The post-electroporation conductivity considered was within the β-dispersion frequency range (1 kHz–100 MHz) [38,39].

The electrical and thermal properties of the metallic electrodes and stent were assumed to remain constant throughout the simulations. Although the electrical conductivity, thermal conductivity, and specific heat of metals are known to vary approximately linearly with temperature over the temperature range encountered in biomedical applications [40], the corresponding changes between physiological temperature and the maximum temperatures predicted in this study are only a few percent. Moreover, the electrical and thermal conductivities of metallic materials remain several orders of magnitude greater than those of the surrounding biological tissues (approximately 10^6^ S/m vs. 0.04–0.7 S/m for electrical conductivity, and ~10 W/m·K vs. 0.2–0.6 W/m·K for thermal conductivity, respectively). Consequently, these variations are expected to have a negligible influence on the electric field distribution, heat transfer, and overall simulation results. For this reason, temperature-dependent metal properties were not included in the model.

The PFA threshold was set to 1000 V/cm for all evaluated tissues. This value was selected based on previously reported in vitro evidence [39], which demonstrated significant loss of viability in neuronal cells and atrial cardiomyocytes exposed to electric fields of approximately 1000 V/cm using monophasic pulses of 100 µs duration at 1 Hz, particularly for pulse number ≥ 30. These pulse characteristics are consistent with the protocol used in the present study. It should be noted, however, that irreversible electroporation thresholds are not universal and depend on several factors, including tissue type, cell morphology, pulse waveform, pulse duration, pulse number, and treatment protocol. Accordingly, the selected threshold should be interpreted as an approximation suitable for the pulse conditions investigated rather than a universal value applicable to all tissues. Supporting this consideration, another in vitro study using both monophasic and biphasic 100 µs pulses reported that cardiac fibroblasts and cardiomyocytes may require higher electric field thresholds (approximately 1250 V/cm, typically with ≥50 pulses at 1 Hz) to achieve comparable levels of cell death [41]. Consequently, the use of a 1000 V/cm threshold in our model likely provides a conservative estimation of the PFA-affected volume.

### 2.4. Boundary Conditions

Electrical, thermal, and fluid dynamics boundary conditions were applied to the outer surfaces of the model, except for those defined by the symmetry plane, which was treated according to symmetry constraints (see Figure 2). Regarding electrical boundary conditions, the PFA protocol was simulated using a train of 60 monophasic pulses of 100 μs at 1 Hz, consistent with protocols reported in preclinical studies for epicardial PFA [17,19]. Electrical energy was delivered using monopolar configuration, between the four metal electrodes of the PFA catheter (acting as active electrodes) and a dispersive electrode (electrical ground condition of 0 V) positioned on all external surfaces of the model, except for the upper surface of the saline layer, which was electrically insulated. Simulations were performed using pulse voltage amplitudes of 1000 V, 2000 V and 2500 V.

For the thermal boundary conditions, all outer surfaces of the model were assumed to be thermally insulated, except for the saline surface, which was defined as a convective heat flux boundary to represent heat exchange with ambient air at 21 °C. A heat transfer coefficient of 20 W/m^2^·K was applied at the air–saline interface [14,30].

For the fluid dynamics boundary conditions, a velocity inlet boundary condition was applied on the inner luminal surface of the LCx coronary artery, enforcing a unidirectional flow (along the *x*-axis) with a velocity of 0.5 m/s, consistent with values reported in the literature [42]. An outlet boundary condition of zero pressure was fixed on the opposite luminal surface of the LCx, enabling the establishment of a pressure-driven flow regime. Within the cardiac cavity, blood flow was modeled in a similar fashion. A velocity inlet boundary condition was applied to the left surface of the cavity’s blood volume, with an imposed flow velocity of 24.4 cm/s [43] in the x-direction. A zero-pressure outlet boundary condition was applied to the right surface of the blood domain. For the saline irrigation system, an inlet flow rate boundary condition was defined and distributed equally across the four infusion ports located within the epicardial PFA catheter’s electrodes, yielding a total saline flow rate of 2 mL/min, as in preclinical studies with the Finger device [17]. The remaining lateral surfaces were assigned zero-pressure outlet boundary conditions, facilitating flow egress and pressure stabilization.

### 2.5. Analyzed Outcomes

The input variables analyzed included energy dosing (i.e., different pulse voltage amplitudes), as well as the relative position and orientation of the intracoronary stent with respect to the metal electrodes of the catheter. The main output variables analyzed were: (1) the PFA-induced lesion size in the target tissue (epicardial fat), serving as an indicator of treatment efficacy; (2) the volume of myocardium and arterial wall affected by PFA, reflecting potential undesired side effects; and (3) any possible collateral thermal damage in the tissues involved (epicardial fat, arterial wall and myocardium).

To evaluate the PFA-induced lesion size in the target, we calculated the volume of epicardial fat exposed to electric field values exceeding 1000 V/cm [44]. Similarly, potential PFA-induced collateral lesions in the myocardium and the arterial wall were assessed by quantifying the tissue volume exposed to electric field values above 1000 V/cm. PFA-induced thermal damage, if present, was also evaluated. To this end, simulations included a 90 s post-PFA latency period to account for the effects of thermal latency and their impact on the progression of tissue thermal damage [33]. This was achieved by monitoring the maximum temperature evolution and temperature distribution in the fat, myocardium, and arterial wall. The extent of thermal damage was determined using the Arrhenius equation (Equation (8)), which relates the rate of thermal damage accumulation to temperature as follows:

(8) $$\Omega (T,t)=A\int_{0}^{t}e^{-\frac{\Delta E}{R\;T(\tau )}}\;d\tau$$

where *A* is the frequency factor (7.39 × 10^39^ s^−1^) and Δ*E* is the activation energy (2.557 × 10^5^ J/mol) [33]. We considered Ω = 1 as the thermal lesion contour, which represents 63% of dead cells [45].

### 2.6. Verification of the Model

Model verification was conducted to determine the optimal outer dimensions (X and Y) as in [30]. The value of the volume of tissue affected by PFA was considered as a control parameter. To determine these dimensions, we increased their values by equal amounts, and when the difference in volume between consecutive simulations was less than 1%, we considered the former values to be adequate. We then determined the adequate spatial resolution by means of similar analysis using the same control parameter. Discretization was spatially heterogeneous, where the finest zone was the electrode–tissue interface since the largest voltage gradient was produced and hence the maximum value of current density. In the tissue, grid size increased gradually with distance from the electrode–tissue interface. This verification analysis provided X = 80 mm and Y = 40 mm of the outer dimensions.

### 2.7. Data Analysis

A total of 27 simulations were conducted to compare the PFA-induced tissue volume between the artery and stented-artery conditions for both artery orientations relative to the catheter (parallel and perpendicular). PFA-induced lesion volumes were evaluated from the final five pulses after the computed PFA-induced lesion volume had stabilized. Because the computational model is deterministic, no inferential statistical analysis was performed. Results are therefore presented as direct comparisons between the different simulation conditions.

## 3. Results

### 3.1. Effect of Stented and Non-Stented Arteries on PFA-Induced Lesion Volume

**Figure 4:**
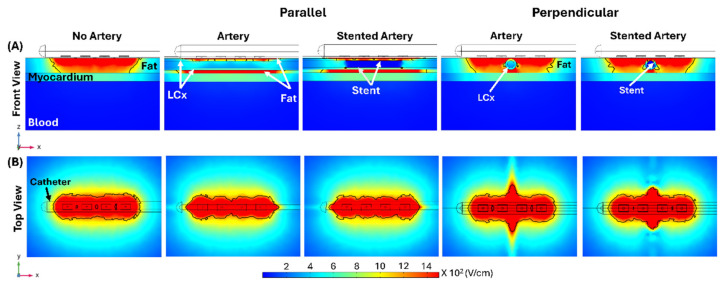
Distribution of the electric field ((**A**): front view—XZ plane; (**B**): top view—XY plane) for the three scenarios considered (no artery, artery, and stented artery) using 1000 V pulses with the artery positioned parallel and perpendicular to the catheter. The black contour line represents the region where the electric field is ≥1000 V/cm, corresponding to the PFA-induced lethal threshold.

**Figure 5:**
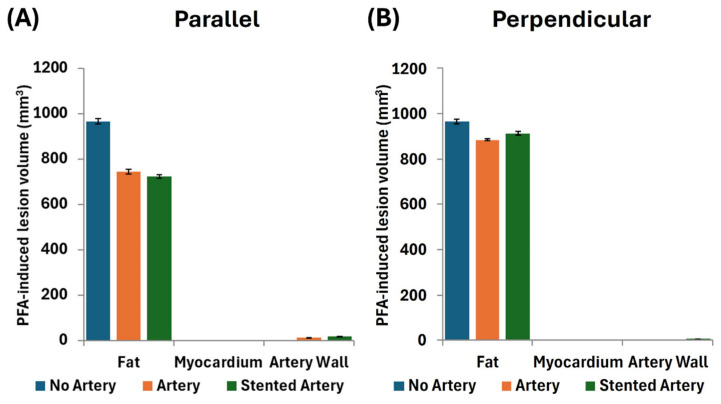
Volume of fat, myocardium and the artery wall within the region where the electric field is ≥1000 V/cm (the threshold for the PFA-induced lethal threshold) for both the parallel (**A**) and the perpendicular (**B**) artery orientation. All three scenarios studied were included: no artery, artery, and stented artery.

Figure 4 shows the electric field distribution across the three scenarios studied (no artery, artery and stented artery) with the artery placed parallel and perpendicular to the catheter, considering the pulse voltage of 1000 V used in clinics. Front (XZ plane) and top (XY plane) views are provided for each scenario. The black contour line delineates regions where the electric field strength is ≥ 1000 V/cm, representing the PFA-induced lethal threshold. The volumes of fat, myocardium and the artery wall within this 1000 V/cm boundary are quantified in Figure 5.

As shown in the figures, the volume of epicardial fat exposed to PFA (electric field strength ≥ 1000 V/cm) was largest when no artery was present (965 mm^3^). In contrast, the presence of an artery reduced the PFA-exposed fat volume, with a more pronounced reduction observed in parallel orientation (744 mm^3^ and 885 mm^3^ for parallel and perpendicular orientations, respectively). This decrease under parallel alignment can be partially explained by the physical space occupied by the artery itself (770 mm^3^), which limits the amount of epicardial fat available within the target region. The simulations predicted a lower PFA-induced fat volume in the stented artery compared with the artery case in the parallel configuration, whereas a slight increase was observed for the perpendicular configuration. In all three scenarios (no artery, artery, and stented artery), the PFA-induced volume appears to be confined to the epicardial fat tissue (target), thereby preserving the underlying myocardium.

**Figure 6:**
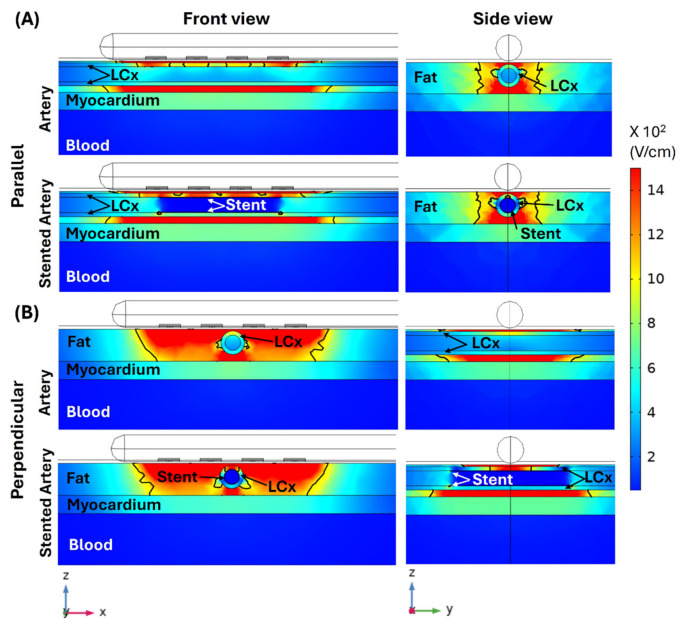
Detailed front (XZ plane) and side (YZ plane) views of the electric field distribution with the artery oriented parallel (**A**) and perpendicular (**B**) to the catheter, in both non-stented and stented conditions, using 1000 V pulses. The black contour line delineates the region where the electric field is ≥1000 V/cm, corresponding to the PFA-lethal threshold.

**Figure 7:**
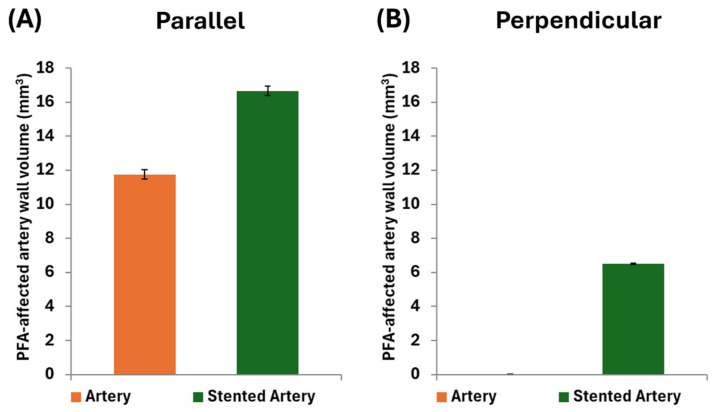
Volume of artery wall affected by PFA with the artery oriented parallel (**A**) and perpendicular (**B**) to the catheter.

Although no PFA-induced myocardial effects were detected under any of the scenarios evaluated, localized electric field enhancement occurred at the arterial wall, particularly in the parallel orientation (Figure 5A). To further investigate this observation, a detailed analysis was conducted. Figure 6 shows detailed front and side views of the electric field around the artery wall for both stented and non-stented arteries in parallel and perpendicular orientations. These side views correspond to the YZ-plane, located midway between the two central electrodes, and were enlarged around the critical treatment zone to enhance visualization. The quantification of the PFA-induced arterial wall volume is represented in Figure 7. PFA-induced electric field enhancement at the arterial wall was more pronounced in the presence of a stent. Notably, in the perpendicular configuration, regions of increased field exposure within the artery wall volume were observed exclusively when a stent was present (see side view in Figure 6B). In the parallel orientation, the stented artery exhibited dramatically higher arterial wall PFA-exposure compared with the non-stented artery. A similar increase was observed in the perpendicular orientation, reflecting the strong local amplification of the electric field induced by the metallic stent.

As Figure 7 shows, the stented artery in the perpendicular orientation exhibited a smaller PFA-affected arterial wall volume compared to the parallel orientation (6.4 mm^3^ vs. 16.6 mm^3^, corresponding to 1.46% and 3.83% of the total arterial volume, respectively). Conversely, in the absence of a stent, arterial wall exposure to PFA was negligible under perpendicular alignment, whereas a measurable volume above the PFA-threshold persisted in the parallel configuration (0 mm^3^ vs. 10.6 mm^3^, corresponding to 0% and 2.4% of the total arterial volume, respectively).

To determine the minimum safety distance required to eliminate the stent’s amplifying effect on the arterial wall, we evaluated several configurations in which the stent was progressively displaced by an offset distance (*d*) from its initial position directly beneath the two central metal electrodes of the catheter toward the left edge of the model (as shown in Figure 2). This analysis was conducted exclusively in parallel orientation, previously identified as the configuration most affected by the presence of the stent. The results indicated that the PFA-affected arterial wall volume became comparable to that observed in the non-stented artery condition when *d* reached a minimum value of 27 mm.

### 3.2. Effect of Stented and Non-Stented Arteries on PFA Thermal Response

**Figure 8:**
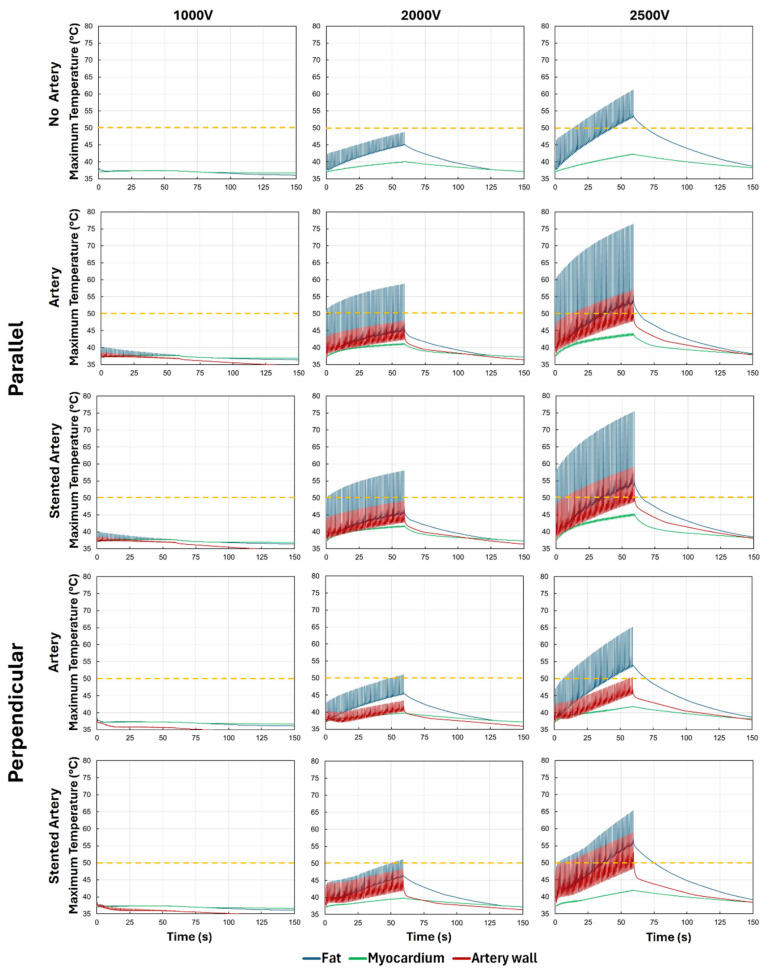
Maximum temperature evolution for the three tested scenarios (no artery, artery and stented artery) with the artery in parallel and perpendicular positions relative to the catheter. Simulations were conducted using applied voltages of 1000, 2000 and 2500 V, measured after the 60 s PFA procedure followed by a 90 s post-latency period. Thermal damage starts with temperatures greater than 50 °C (yellow dashed line).

Figure 8 shows the progression of the maximum temperature in the different tissues involved (fat, arterial wall, and myocardium) across the three scenarios tested (no artery, artery and stented artery) and voltage settings (1000, 2000 and 2500 V). Temperature evolution was also monitored for an extra 90 s after PFA pulse delivery, to account for thermal latency. Both parallel and perpendicular orientations of the artery relative to the catheter were considered.

Under standard clinical treatment conditions (1000 V pulses), no appreciable thermal effects were observed in any of the tissues or artery configurations evaluated, with maximum temperatures remaining below 40 °C (i.e., thermal damage starts with temperatures greater than 50 °C). Noticeable temperature increases occurred only when the pulse voltage exceeded 2000 V, affecting the epicardial fat (target region). The myocardium remained thermally unaffected in all cases, exhibiting maximum temperatures consistently below 45 °C.

As illustrated in Figure 8, both orientations followed similar thermal trends, showing a progressive rise in temperature with increasing applied voltage. However, clear differences were observed between configurations. In the parallel orientation, the fat tissue exhibited higher peak temperatures once the applied voltage exceeded 2000 V, reaching values close to 60 °C, compared with approximately 50 °C in the perpendicular orientation. At higher voltages, heating above 50 °C extended to the arterial wall region; nonetheless, this occurred in the perpendicular orientation only when a stent was present, whereas in the parallel configuration, similar temperature levels were reached both with and without the stent.

These observations highlight the distinct influence of stent presence and artery orientation on local heating. The presence of a metallic stent did not substantially alter the temperature distribution in the surrounding fat; however, it produced a measurable increase in the arterial wall temperature, particularly pronounced in the perpendicular orientation. This observation is consistent with the previously discussed amplification of the local electric field intensity at the arterial wall due to the presence of the metallic stent.

**Figure 9:**
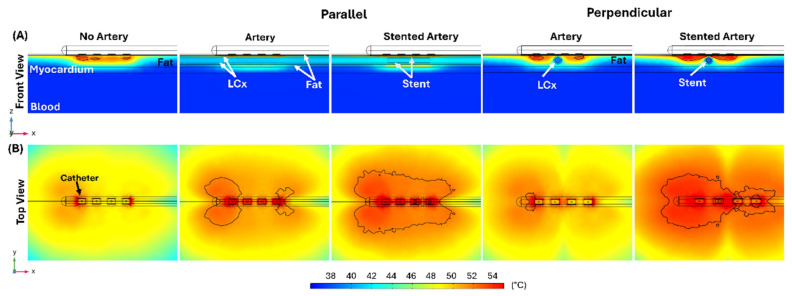
Temperature distribution at the end of PFA using 2500 V pulses for the different scenarios (no artery, artery and stented artery) studied with the artery placed parallel and perpendicular to the catheter. Panels (**A**) and (**B**) show the front (XZ plane) and the top (XY plane) views respectively. The solid black line represents the thermal damage contour Ω = 1.

This effect is more clearly illustrated in Figure 9, which depicts the temperature distribution and the thermal damage volume (outlined in solid black) after the final PFA application (60 s) using 2500 V pulses (representing the critical voltage for thermal heating). The results are shown for the three scenarios (no artery, artery and stented artery) with both parallel and perpendicular artery orientations relative to the catheter.

The presence of the stent within the artery led to an increased final temperature in the fat, resulting in a marked expansion of the thermal damage area, as evident in the top view images for both orientations. Specifically, the stented artery was associated with an increased thermal damage volume, from 68 to 228 mm^3^ in the parallel orientation and from 45 to 282 mm^3^ in the perpendicular orientation. This corresponds to an increase in the thermal damage volume of 235 and 527% for the parallel and perpendicular configurations, respectively.

**Figure 10:**
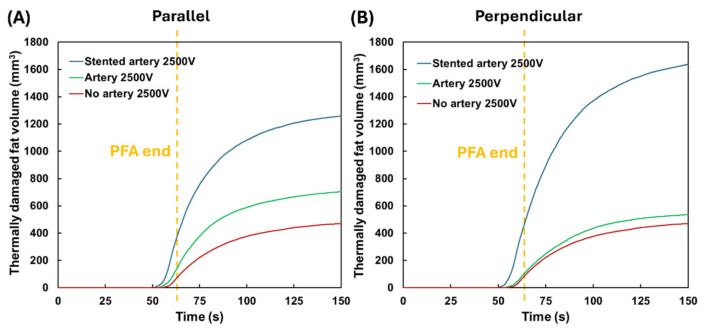
Temporal evolution of the thermally damaged fat volume, accounting for a 90 s latency period following the 60 s PFA application using 2500 V, for both artery orientations: parallel (**A**) and perpendicular (**B**) relative to the catheter. Only damage within the fat tissue is shown, as no thermally induced cell death was detected in other tissues (myocardium or arterial wall) under the evaluated conditions.

Given that a substantial temperature rise and an extension of the thermal damage have been observed at the end of the PFA application using 2500 V, it is therefore crucial to evaluate whether the thermal damage volume continues to increase within the epicardial fat during the 90 s latency period (i.e., the interval without PFA energy delivery). Figure 10 illustrates the temporal evolution of thermal damage in the epicardial fat tissue throughout this latency period. No thermal damage was detected in either the arterial wall or the myocardium.

As shown in Figure 10, a marked rise in thermally induced lesion volume was observed during the 90 s latency period, particularly in the presence of a stented artery, indicating a pronounced thermal latency effect. The comparison between artery orientations (Figure 10A,B) reveals that the perpendicular configuration produced a greater and more sustained increase in thermal damage volume compared with the parallel orientation. This difference was especially evident for the stented artery case, where heat accumulation was enhanced due to the metallic stent, leading to higher peak temperatures and larger final lesion volumes.

**Figure 11:**
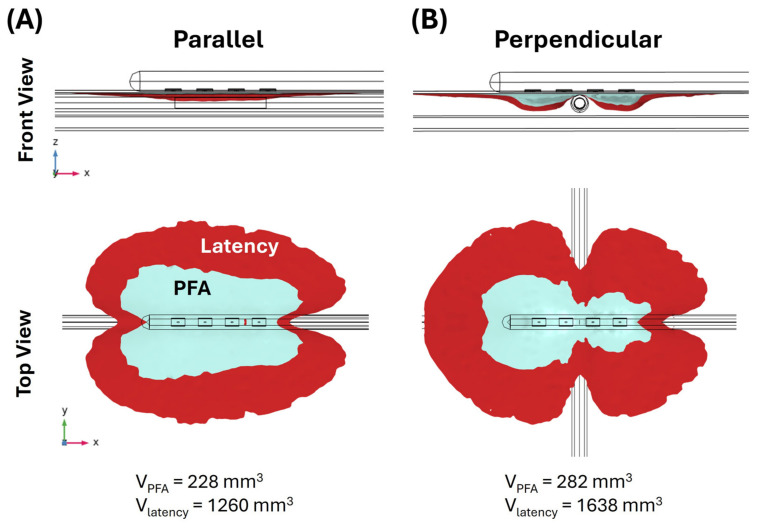
Detail of front (XZ plane) and top (XY plane) views of the fat volume thermally damaged just after PFA (in light green color) and after the latency period (in red color) for the case of 2500 V pulses with a stented artery in parallel (**A**) and perpendicular (**B**) positions with respect to the catheter.

To better visualize the spatial progression of the thermal damage observed in Figure 10, Figure 11 provides a detailed comparison of the fat tissue volume affected immediately after PFA (light green) and after the 90 s latency period (red). This representation highlights the post-PFA expansion of the thermally damaged region within the epicardial fat, which was more pronounced in the perpendicular orientation. Quantitatively, the thermally damaged fat volume increased from 228 mm^3^ to 1260 mm^3^ in the parallel configuration and from 282 mm^3^ to 1638 mm^3^ in the perpendicular configuration after the latency period, representing an increase of approximately 453% and 481%, respectively. These findings confirm the strong influence of the metallic stent in enhancing heat retention within the epicardial fat and demonstrate the significant contribution of thermal latency to thermal lesion expansion following high-voltage PFA application.

## 4. Discussion

The use of ablation techniques, particularly pulsed field ablation (PFA), for the treatment of cardiac arrhythmias is well established and has recently emerged as an alternative option for the selective targeting of ganglionated plexi (GPs) from an epicardial approach. Existing computational studies [14,15,16] have provided useful preliminary insights, but their methodological simplifications including non-PFA-specific catheter designs, limited representation of blood-flow-related thermal interactions, and non-clinical pulse protocols restrict their ability to reproduce the biophysical conditions encountered in vivo. To address these limitations, the present study introduces a 3D multiphysics PFA model simulating the most critical scenario, in which the catheter is positioned on the epicardial surface in close proximity to a stented coronary artery. The model incorporates clinically representative PFA parameters, an accurate PFA catheter (Finger device, AtriAN Medical, Galway, Ireland), and a fully coupled electrical–thermal–fluid dynamic formulation with explicit consideration of post-treatment thermal latency. This framework provides a more physiologically realistic basis for evaluating PFA performance in anatomically complex settings, particularly those involving coronary arteries and metallic stents.

The results demonstrated that under epicardial PFA treatment parameters (1000 V, 60 pulses of 100 µs duration with 1 s inter-pulse intervals), and using the custom-designed Finger device catheter, the electric field distribution, and consequently the PFA-induced volume, was largely confined to the epicardial fat. This confirms the high tissue selectivity of PFA and the absence of myocardial collateral damage. These findings were consistent regardless of whether an artery, stented or non-stented, was located in close proximity to the PFA catheter, and for both parallel and perpendicular orientations of the artery relative to the catheter. This outcome aligns with preclinical and clinical evidence demonstrating preferential epicardial PFA of ganglionated plexi while preserving the underlying myocardium [6,7].

Although PFA is characterized by its non-thermal selectivity, several recent investigations have demonstrated that coronary vasospasm may occur when ablation is performed near epicardial vessels, including the right coronary and circumflex arteries. Preclinical studies in swine have shown that both intracoronary and epicardial PFA can produce transient vessel narrowing in nearly half of all applications, with spontaneous or nitrate-induced resolution and minimal chronic damage [9]. Clinical observations using intracoronary imaging confirm similar behavior: transient luminal constriction and mild late narrowing have been documented after cavotricuspid and mitral isthmus ablation [10,11,46]. Moreover, although severe events are rare, isolated cases of diffuse or multivessel spasm following extensive PFA delivery have been reported [47]. These converging findings indicate that vasospasm represents a class effect of PFA energy rather than a device-specific artifact [12].

Previous modeling studies [14,15,16] reported electric field perturbations in the vicinity of metallic stents, but the simplified nature of their computational formulations limited their ability to spatially and quantitatively characterize this phenomenon. By incorporating an epicardial PFA-specific catheter, clinically representative PFA pulse parameters and vessel geometries together with fully coupled electrical–thermal–fluid dynamics, the present model resolves these interactions with substantially higher fidelity. The simulations show that electric field amplification occurs primarily at the arterial wall–fat interface in the presence of a stent, although the PFA-induced volume of arterial wall tissue exposed to elevated field magnitudes remains limited under clinically applied voltages.

In this context, the present computational study offers a complementary, mechanistic insight. By deliberately simulating a worst-case configuration, a metallic stent directly beneath the PFA electrodes and a minimal electrode–artery distance, the model enables assessment of how strong electric fields may concentrate near conductive vascular structures. This approach provides a controlled environment to explore whether field intensification or local current redistribution could contribute to smooth-muscle depolarization and transient vasoconstriction. The results demonstrate that, at clinically relevant voltages, the stent predominantly acts as a passive shield without significant amplification of the perivascular electric field, suggesting that vasospasm is more likely due to direct electroporation-induced activation of vascular smooth muscle cells rather than to geometric field enhancement.

In addition, the model enables the derivation of a 27 mm electrode–stent offset distance as a quantitative geometric parameter for characterizing stent–field interactions. This parameter provides a spatial reference for evaluating electrode placement in anatomical configurations with limited catheter–artery separation, thereby improving the computational assessment of epicardial PFA in scenarios involving pre-existing coronary stents. Consequently, the modeling framework developed here can serve as a tool to interpret and contextualize potential coronary artery wall damage events during epicardial PFA and to establish safer energy dosing and catheter positioning guidelines.

Unlike previous reports, the present study incorporated convective heat transfer from blood flow and accounted for thermal latency, allowing for a more clinically relevant analysis of temperature evolution during and after PFA. Under these conditions, the results showed that clinically relevant voltages (1000 V) did not produce any thermal effects, while higher voltages (≥2000 V) led to measurable temperature elevations, primarily confined to epicardial fat. Importantly, myocardial temperatures remained always below 45 °C across all tested scenarios. These findings suggest that, in contrast to previous studies based on static thermal assumptions, the risk of excessive heating during epicardial PFA is lower than previously anticipated under typical treatment conditions. Additionally, the simulations highlight the critical protective role of blood perfusion in dissipating localized heat and limiting thermal damage.

A key contribution of this study is the quantification of post-procedural heat accumulation due to thermal latency. Following high-voltage application (2500 V), the thermally affected fat volume increased more than fourfold during the 90 s post-ablation period. This represents the first known demonstration of delayed thermal lesion expansion in PFA, a phenomenon previously described only in radiofrequency ablation models [33]. These results underscore that, although PFA is primarily regarded as a non-thermal ablation modality, secondary thermal effects can become clinically significant at elevated voltages, and more relevant in the presence of metallic structures. This finding highlights the importance of extending computational analyses beyond the PFA pulse protocol to capture delayed heat transfer phenomena that may contribute to tissue damage.

Despite the novel and valuable insights provided by this study, several limitations should be acknowledged. Firstly, although direct experimental validation of the model is still required, there is currently a lack of experimental and clinical studies specifically evaluating electrical and thermal interactions near stented coronary arteries during epicardial PFA under clinically relevant conditions, which limits the availability of data for direct model validation [48]. Nevertheless, the modeling framework employed is based on established methodologies that have been widely used in previous studies of PFA [18,36,49,50,51], supporting the assessment of how coronary stents influence electrical and thermal responses during epicardial PFA. Secondly, the model assumes isotropic and homogeneous tissue properties, whereas biological tissues, particularly myocardium and fat, exhibit anisotropy and heterogeneity that may influence the local electric field distribution. Thirdly, the analysis was limited to a single catheter design (the Finger device) and a specific stent material (stainless steel), while alternative commercial catheter configurations or stent compositions could produce somewhat different electrical and thermal responses. In particular, cobalt-chromium and nitinol stents, which are widely used in clinical practice, have electrical conductivities on the order of 10^6^ S/m and thermal conductivities of approximately 10–11 W/m·K [52], compared with 7.4 × 10^6^ S/m and 15 W/m·K for the stainless steel considered in this study. Therefore, these materials remain within the same order of magnitude as stainless steel, with electrical and thermal conductivities substantially higher than those of the surrounding biological tissues (0.04–0.7 S/m and 0.2–0.6 W/m·K, respectively). Consequently, although quantitative differences in local electric field enhancement and temperature distribution may occur, substantial changes in the overall electrical and thermal response are not expected. In addition, cardiac motion and dynamic catheter–tissue contact were not included, which may affect transient electrical and thermal behavior during in vivo application.

Another limitation concerns the use of a single irreversible electroporation threshold (1000 V/cm) for all evaluated tissues. Although this value was selected based on experimental studies using pulse parameters comparable to those employed in the present work [18,36,49,50,51] and therefore represents a reasonable approximation for neuronal cells and atrial cardiomyocytes, electroporation thresholds are known to depend on cell type, tissue microstructure, waveform, pulse duration, pulse number, and pulse polarity. Consequently, the absolute volumes predicted to be affected by PFA, particularly within the arterial wall, should be interpreted as threshold-dependent estimates rather than exact biological PFA-induced lesion volumes. Nevertheless, the primary objective of this study was to compare the relative influence of artery orientation and stent presence under identical modeling conditions. Therefore, while the absolute affected volumes may vary if different tissue-specific thresholds are considered, the relative trends and the observed localized electric field enhancement at the arterial wall are expected to remain unchanged.

Finally, although recent experimental evidence has demonstrated that PFA-induced coronary vasospasm is strongly associated with local electric field intensity and electroporation of the tunica intima [53], a direct quantitative correlation between electric field magnitude and vasospasm onset has not yet been established. Consequently, there are currently no experimental data allowing the definition of a specific E-field threshold above which vasospasm occurs. In this regard, the present study demonstrated that the presence of a metallic stent can locally amplify the electric field at the arterial wall, increasing the PFA-affected arterial wall volume up to 3.83% in the worst-case configuration. However, once such threshold data become available, the multiphysics model developed here could be used to predict under which conditions (e.g., pulse protocol, catheter-to-artery distance, or stent position) vasospasm would be expected to occur.

Although the present computational model provides valuable insights into the electrical and thermal interactions during epicardial PFA near coronary arteries, further experimental, clinical, and long-term studies are required to fully establish the safety and reliability of the technique, particularly as its clinical application expands to younger patient populations. Future work should also incorporate patient-specific geometries derived from medical imaging, additional stent materials to represent a broader range of clinical scenarios, and experimental platforms capable of reproducing PFA delivery near stented coronary arteries under controlled conditions to validate the electrical and thermal interactions predicted by computational models. In addition, investigating alternative PFA pulse trains and multi-electrode energy delivery configurations may help optimize treatment protocols by improving the balance between tissue selectivity and procedural safety under clinically realistic conditions.

## 5. Conclusions

Our findings suggest that under clinical conditions (1000 V, 60 × 100 µs pulses at 1 Hz), electric fields remain confined to epicardial fat, preserving myocardial tissue regardless of artery orientation or stent implantation, supporting the intrinsic selectivity of PFA. Although metallic stents locally perturb electric field distributions and induce collateral electrical effects in the arterial wall, these effects remain spatially confined and involve at most 3.83% of the arterial wall volume in the worst-case configuration. Thermal analyses incorporating blood perfusion show negligible heating at clinical voltages and confirm the protective role of convective cooling. However, at higher voltages (≥2500 V), delayed thermal effects can significantly enlarge the affected fat volumes, emphasizing the need to account for thermal latency. Future studies integrating patient-specific anatomies and high-fidelity vascular models may further clarify how electrical parameters and stent properties influence vascular responses, providing a mechanistic framework for evaluating vascular safety and vasospasm risk in PFA-based treatments for cardiac arrhythmias.

## Figures and Tables

**Table 1:** Electrical and thermal properties of the model elements [14,30,31,32,33].

Element/Material	*σ*_0_ (S/m)	*σ*_1_ (S/m)	*Ɛ* *_r_*	*k* (W/m·K)	*ρ* (kg/m^3^)	*C_p_* (J/kg·K)	µ (Pa·s)
Electrode/Pt-Ir	4.6 × 10^6^		1	71	21,500	132	-
Stent/ Stainless Steel	7.4 × 10^6^		1	15	8000	480	-
Catheter/ Polyurethane	10^−5^		3	23	1440	1050	-
Saline	1.392		1	0.628	980	4184	0.95 × 10^−3^
Epicardial fat/ Adipose tissue	0.0377	0.0438	56.8	0.21	911	2348	-
Heart/myocardium	0.0537	0.281	3260	0.56	1081	3686	-
Cardiac chamber/ Blood	0.7		5260	0.52	1050	3617	3.3 × 10^−3^
Coronary artery/ Blood vessel wall	0.251	0.324	312	0.46	1102	3306	-

*σ*: Electrical conductivity ($\sigma_{0}$ and $\sigma_{1}$ are the pre- and post-electroporation electrical conductivity values, respectively); *k*: thermal conductivity; ρ: density; *Cp*: specific heat; µ: dynamic viscosity.

## Data Availability

The original contributions presented in this study are included in the article. Further inquiries can be directed to the corresponding author.

## Abbreviations

The following abbreviations are used in this manuscript:

PFA	Pulsed field ablation
GPs	Ganglionated plexi
LCx	Left circumflex coronary artery

## References

[1] Hindricks G., Potpara T., Dagres N., Arbelo E., Bax J.J., Blomström-Lundqvist C., Boriani G., Castella M., Dan G.-A., Dilaveris P.E. (2021). 2020 ESC Guidelines for the Diagnosis and Management of Atrial Fibrillation Developed in Collaboration with the European Association for Cardio-Thoracic Surgery (EACTS). Eur. Heart J..

[2] Kuck K.-H., Hoffmann B.A., Ernst S., Wegscheider K., Treszl A., Metzner A., Eckardt L., Lewalter T., Breithardt G., Willems S. (2016). Impact of Complete Versus Incomplete Circumferential Lines Around the Pulmonary Veins During Catheter Ablation of Paroxysmal Atrial Fibrillation. Circ. Arrhythm. Electrophysiol..

[3] Berger W.R., Meulendijks E.R., Limpens J., van den Berg N.W.E., Neefs J., Driessen A.H.G., Krul S.P.J., van Boven W.J.P., de Groot J.R. (2019). Persistent Atrial Fibrillation: A Systematic Review and Meta-Analysis of Invasive Strategies. Int. J. Cardiol..

[4] Sugrue A., Vaidya V., Witt C., DeSimone C.V., Yasin O., Maor E., Killu A.M., Kapa S., McLeod C.J., Miklavčič D. (2019). Irreversible Electroporation for Catheter-Based Cardiac Ablation: A Systematic Review of the Preclinical Experience. J. Interv. Card. Electrophysiol..

[5] Wittkampf F.H.M., van Es R., Neven K. (2018). Electroporation and Its Relevance for Cardiac Catheter Ablation. JACC Clin. Electrophysiol..

[6] O’Brien B., Reilly J., Coffey K., González-Suárez A., Quinlan L., van Zyl M. (2023). Cardioneuroablation Using Epicardial Pulsed Field Ablation for the Treatment of Atrial Fibrillation. J. Cardiovasc. Dev. Dis..

[7] Musikantow D.R., Reddy V.Y., Skalsky I., Shaburishvili T., van Zyl M., O’Brien B., Coffey K., Reilly J., Neuzil P., Asirvatham S. (2025). Targeted Ablation of Epicardial Ganglionated Plexi during Cardiac Surgery with Pulsed Field Electroporation (NEURAL AF). J. Interv. Card. Electrophysiol..

[8] Musikantow D.R., Reddy V.Y., Shaburishvili T., van Zyl M., O’Brien B., Coffey K., Reilly J., Asirvatham S., de Groot J.R. (2024). Epicardial Pulsed Field Ablation for the Treatment of Paroxysmal Atrial Fibrillation During Cardiac Surgery. JACC Clin. Electrophysiol..

[9] Ladejobi A., Christopoulos G., Tan N., Ladas T.P., Tri J., van Zyl M., Yasin O., Sugrue A., Khabsa M., Uecker D.R. (2022). Effects of Pulsed Electric Fields on the Coronary Arteries in Swine. Circ. Arrhythm. Electrophysiol..

[10] Tam M.T.K., Chan J.Y.S., Chan C.P., Wu E.B., Lai A., Au A.C.K., Chi W.K., Tan G., Yan B.P. (2025). Effect of Pulsed-Field Ablation on Human Coronary Arteries. JACC Clin. Electrophysiol..

[11] Malyshev Y., Neuzil P., Petru J., Funasako M., Hala P., Kopriva K., Schneider C., Achyutha A., Vanderper A., Musikantow D. (2024). Nitroglycerin to Ameliorate Coronary Artery Spasm During Focal Pulsed-Field Ablation for Atrial Fibrillation. JACC Clin. Electrophysiol..

[12] Ramirez D.A., Garrott K., Garlitski A., Koop B. (2024). Coronary Spasm Due to Pulsed Field Ablation: A State-of-the-Art Review. Pacing Clin. Electrophysiol..

[13] Farnir F.I.P., Chaldoupi S.-M., Hermans B., Johannessen A., Haugdal M.A., Ruwald M.H., Mohaissen T., Saljic A., Jerltorp K., Nissen S.D. (2026). Ablation of Cavotricuspid Isthmus–Dependent Atrial Flutter Using a Focal Monopolar Pulsed-Field Ablation Catheter: Feasibility, Periprocedural Coronary Spasms and Conduction Disorders. Heart Rhythm.

[14] González-Suárez A., Pérez J.J., O’Brien B., Elahi A. (2022). In Silico Modelling to Assess the Electrical and Thermal Disturbance Provoked by a Metal Intracoronary Stent during Epicardial Pulsed Electric Field Ablation. J. Cardiovasc. Dev. Dis..

[15] Wang Z., Liang M., Sun J., Zhang J., Li Y., Xu L., Han Y. (2024). Epicardial Pulsed-Field Ablation-Impact of Electric Field and Heat Distribution Induced by Coronary Metallic Stents. Front. Cardiovasc. Med..

[16] Wang Z., Liang M., Sun J., Zhang J., Li Y., Zhang D., Han Y. (2025). Effects of Electric Field and Heat Distribution Due to Trends in Metal Stent Diameter in Epicardial Pulsed Field Ablation. Sci. Rep..

[17] van Zyl M., Khabsa M., Tri J., Ladas T., Yasin O., Ladejobi A., Reilly J., O’Brien B., Coffey K., Asirvatham S. (2022). Open-Chest Pulsed Electric Field Ablation of Cardiac Ganglionated Plexi in Acute Canine Models. J. Innov. Card. Rhythm. Manag..

[18] González-Suárez A., Irastorza R.M., Deane S., O’Brien B., O’Halloran M., Elahi A. (2022). Full Torso and Limited-Domain Computer Models for Epicardial Pulsed Electric Field Ablation. Comput. Methods Programs Biomed..

[19] Padmanabhan D., Naksuk N., Killu A.K., Kapa S., Witt C., Sugrue A., DeSimone C.V., Madhavan M., de Groot J.R., O’Brien B. (2019). Electroporation of Epicardial Autonomic Ganglia: Safety and Efficacy in Medium-term Canine Models. J. Cardiovasc. Electrophysiol..

[20] Pauza D.H., Skripka V., Pauziene N., Stropus R. (2000). Morphology, Distribution, and Variability of the Epicardiac Neural Ganglionated Subplexuses in the Human Heart. Anat. Rec..

[21] Yan S., Gu K., Wu X., Wang W. (2020). Computer Simulation Study on the Effect of Electrode–Tissue Contact Force on Thermal Lesion Size in Cardiac Radiofrequency Ablation. Int. J. Hyperth..

[22] Sánchez-Quintana D., López-Mínguez J.R., Macías Y., Cabrera J.A., Saremi F. (2014). Left Atrial Anatomy Relevant to Catheter Ablation. Cardiol. Res. Pract..

[23] González-Suárez A., O’Brien B., O’Halloran M., Elahi A. (2022). Pulsed Electric Field Ablation of Epicardial Autonomic Ganglia: Computer Analysis of Monopolar Electric Field across the Tissues Involved. Bioengineering.

[24] Yokokawa M., Sundaram B., Garg A., Stojanovska J., Oral H., Morady F., Chugh A. (2011). Impact of Mitral Isthmus Anatomy on the Likelihood of Achieving Linear Block in Patients Undergoing Catheter Ablation of Persistent Atrial Fibrillation. Heart Rhythm.

[25] Garcia-Garcia H.M., Sanz-Sanchez J., Pinilla-Echeverri N., Blanco P.J., Bourantas C., Alfonso F. (2025). Advances in Coronary Imaging of Atherosclerotic Plaques. EuroIntervention.

[26] Al Suwaidi J. (2000). Coronary Artery Stents. JAMA.

[27] Sheehan M.C., Srimathveeravalli G., Prakash P., Srimathveeravalli G. (2022). Pulsed Electric Fields. Principles and Technologies for Electromagnetic Energy Based Therapies.

[28] Berjano E.J. (2006). Theoretical Modeling for Radiofrequency Ablation: State-of-the-Art and Challenges for the Future. Biomed. Eng. Online.

[29] Pérez J.J., González-Suárez A., Berjano E. (2018). Numerical Analysis of Thermal Impact of Intramyocardial Capillary Blood Flow during Radiofrequency Cardiac Ablation. Int. J. Hyperth..

[30] Estevez-Laborí F., O’Brien B., González-Suárez A. (2024). Difference between Endocardial and Epicardial Application of Pulsed Fields for Targeting Epicardial Ganglia: An in-Silico Modelling Study. Comput. Biol. Med..

[31] Hasgall P., Di Gennaro F., Baumgartner C., Neufeld E., Lloyd B., Gosselin M., Payne D., Klingenböck A., Kuster N. IT’IS Database for Thermal and Electromagnetic Parameters of Biological Tissues. https://itis.swiss/virtual-population/tissue-properties/database/alternative-tissue-frequency-chart.

[32] Tungjitkusolmun S., Vorperian V.R., Bhavaraju N., Cao H., Tsai J.-Z., Webster J.G. (2001). Guidelines for Predicting Lesion Size at Common Endocardial Locations during Radio-Frequency Ablation. IEEE Trans. Biomed. Eng..

[33] Irastorza R.M., d’Avila A., Berjano E. (2018). Thermal Latency Adds to Lesion Depth after Application of High-power Short-duration Radiofrequency Energy: Results of a Computer-modeling Study. J. Cardiovasc. Electrophysiol..

[34] Guo F., Deng H., Qian K., Li X. (2022). Characterization of Dispersion and Anisotropic-Conductivity in Tissue Model during Electroporation Pulses. Bioelectrochemistry.

[35] Zhao Y., Bhonsle S., Dong S., Lv Y., Liu H., Safaai-Jazi A., Davalos R.V., Yao C. (2018). Characterization of Conductivity Changes During High-Frequency Irreversible Electroporation for Treatment Planning. IEEE Trans. Biomed. Eng..

[36] Zhao Y., Davalos R.V. (2020). Development of an Endothermic Electrode for Electroporation-Based Therapies: A Simulation Study. Appl. Phys. Lett..

[37] Duck F.A. (2013). Physical Properties of Tissues: A Comprehensive Reference Book.

[38] Aycock K.N., Zhao Y., Lorenzo M.F., Davalos R.V. (2021). A Theoretical Argument for Extended Interpulse Delays in Therapeutic High-Frequency Irreversible Electroporation Treatments. IEEE Trans. Biomed. Eng..

[39] Neal R.E., Millar J.L., Kavnoudias H., Royce P., Rosenfeldt F., Pham A., Smith R., Davalos R.V., Thomson K.R. (2014). In Vivo Characterization and Numerical Simulation of Prostate Properties for Non-Thermal Irreversible Electroporation Ablation. Prostate.

[40] The Engineering ToolBox Stainless Steel-Specific Heat and Thermal Conductivities vs. Temperatures. https://www.engineeringtoolbox.com/stainless-steel-specific-heat-thermal-conductivity-vs-temperature-d_2225.html.

[41] Avazzadeh S., Dehkordi M.H., Owens P., Jalali A., O’Brien B., Coffey K., O’Halloran M., Fernhead H.O., Keane D., Quinlan L.R. (2022). Establishing Electroporation Thresholds for Targeted Cell Specific Cardiac Ablation in a 2D Culture Model. J. Cardiovasc. Electrophysiol..

[42] Haynes W.M. (2017). CRC Handbook of Chemistry and Physics.

[43] Dammers R., Stifft F., Tordoir J.H.M., Hameleers J.M.M., Hoeks A.P.G., Kitslaar P.J.E.H.M. (2003). Shear Stress Depends on Vascular Territory: Comparison between Common Carotid and Brachial Artery. J. Appl. Physiol..

[44] Avazzadeh S., O’Brien B., Coffey K., O’Halloran M., Keane D., Quinlan L.R. (2021). Establishing Irreversible Electroporation Electric Field Potential Threshold in A Suspension In Vitro Model for Cardiac and Neuronal Cells. J. Clin. Med..

[45] Chang I.A. (2010). Considerations for Thermal Injury Analysis for RF Ablation Devices. Open Biomed. Eng. J..

[46] Kouguchi T., Sagawa Y., Murata K., Arai H., Oda A., Kishaba J., Yasui Y., Kato Y., Sasano T., Yamauchi Y. (2025). Pulsed-Field Ablation–Induced Coronary Vasospasm for Atrial Tachycardia Originating From the Lateral Tricuspid Annulus. JACC Case Rep..

[47] Mannion J., Gorry C., Foley B., Tuohy S. (2025). Remote Diffuse Multi-Coronary Artery Spasm Following Pulmonary Vein and Posterior Wall Isolation, Using a Focal Monopolar Pulsed Field Ablation Catheter. Heart Rhythm Case Rep..

[48] Seidabadi L., Vandenbussche I., Carter Fink R., Moore M., McCorkendale B., Esmailie F. (2025). Role of Computational Modelling in Enhancing Thermal Safety During Cardiac Ablation. Interdiscip. Cardiovasc. Thorac. Surg..

[49] Ji X., Zhang H., Zang L., Yan S., Wu X. (2022). The Effect of Discharge Mode on the Distribution of Myocardial Pulsed Electric Field—A Simulation Study for Pulsed Field Ablation of Atrial Fibrillation. J. Cardiovasc. Dev. Dis..

[50] Neal R.E., Garcia P.A., Robertson J.L., Davalos R.V. (2012). Experimental Characterization and Numerical Modeling of Tissue Electrical Conductivity during Pulsed Electric Fields for Irreversible Electroporation Treatment Planning. IEEE Trans. Biomed. Eng..

[51] Wang Z., Li Y., Liang M., Sun J., Zhang J., Xu L., Han Y. (2025). Feasibility Study of Intravascular Pulsed Electric Field Ablation for the Treatment of Cardiac Arrhythmias. Front. Physiol..

[52] MatWeb LLC MatWeb: Online Materials Information Resource. https://www.matweb.com/.

[53] Amorós-Figueras G., Castellví Q., Casabella-Ramon S., Soriano-Amores M., Borachok Á., del Pino P., Moreno-Weidmann Z., Ivorra A., Guerra J.M. (2026). Angiographic and Histological Characterization of Pulsed Field Ablation-Induced Coronary Spasm: Differential Effect of 2 Waveforms. Heart Rhythm.

